# Multi-omic characterization of bifunctional peroxidase 4-coumarate 3-hydroxylase knockdown in *Brachypodium distachyon* provides insights into lignin modification-associated pleiotropic effects

**DOI:** 10.3389/fpls.2022.908649

**Published:** 2022-09-28

**Authors:** Him K. Shrestha, Yosef Fichman, Nancy L. Engle, Timothy J. Tschaplinski, Ron Mittler, Richard A. Dixon, Robert L. Hettich, Jaime Barros, Paul E. Abraham

**Affiliations:** ^1^ Biosciences Division, Oak Ridge National Laboratory, Oak Ridge, TN, United States; ^2^ Genome Science and Technology, University of Tennessee-Knoxville, Knoxville, TN, United States; ^3^ Division of Plant Sciences and Interdisciplinary Plant Group, University of Missouri, Columbia, MO, United States; ^4^ BioDiscovery Institute and Department of Biological Sciences, University of North Texas, Denton, TX, United States

**Keywords:** plant proteomics and functional genomics, redox homeostasis and signaling, lignin modifications, *Brachypodium distachyon*, pleiotrofic effects

## Abstract

A bifunctional peroxidase enzyme, 4-coumarate 3-hydroxylase (C3H/APX), provides a parallel route to the shikimate shunt pathway for the conversion of 4-coumarate to caffeate in the early steps of lignin biosynthesis. Knockdown of C3H/APX (C3H/APX-KD) expression has been shown to reduce the lignin content in *Brachypodium distachyon*. However, like many other lignin-modified plants, C3H/APX-KDs show unpredictable pleiotropic phenotypes, including stunted growth, delayed senescence, and reduced seed yield. A system-wide level understanding of altered biological processes in lignin-modified plants can help pinpoint the lignin-modification associated growth defects to benefit future studies aiming to negate the yield penalty. Here, a multi-omic approach was used to characterize molecular changes resulting from C3H/APX-KD associated lignin modification and negative growth phenotype in *Brachypodium distachyon*. Our findings demonstrate that C3H/APX knockdown in Brachypodium stems substantially alters the abundance of enzymes implicated in the phenylpropanoid biosynthetic pathway and disrupt cellular redox homeostasis. Moreover, it elicits plant defense responses associated with intracellular kinases and phytohormone-based signaling to facilitate growth-defense trade-offs. A deeper understanding along with potential targets to mitigate the pleiotropic phenotypes identified in this study could aid to increase the economic feasibility of lignocellulosic biofuel production.

*Notice: This manuscript has been authored by UT-Battelle, LLC under Contract No. DE-AC05- 00OR22725 with the U.S. Department of Energy. The United States Government retains and the publisher, by accepting the article for publication, acknowledges that the United States Government retains a non-exclusive, paid-up, irrevocable, worldwide license to publish or reproduce the published form of this manuscript, or allow others to do so, for United States Government purposes. The Department of Energy will provide public access to these results of federally sponsored research in accordance with the DOE Public Access Plan (* http://energy.gov/downloads/doe-public-access-plan *).*

## Introduction

Lignin is a natural, branched plant phenolic heteropolymer generated by radical coupling of monomers called monolignols, mainly coniferyl, sinapyl, and *p*-coumaryl alcohols, which creates a gluing matrix for cellulose fibers in higher plant cell walls (Boerjan et al., 2003; Ralph et al., 2004). In general, lignin is a crucial secondary plant cell walls component, providing structural and physical integrity to support plant growth and development, and also functions in plant defense (Whetten and Sederoff, 1995; Boerjan et al., 2003; Zhao and Dixon, 2014). Additionally, lignification benefits plants by preserving cell wall integrity against a range of environmental stressors (Delessert et al., 2004; Hilal et al., 2004; Fan et al., 2006; Malinovsky et al., 2014; Barros et al., 2015; Lee et al., 2019); however, this makes it challenging to access the available cellulosic biomass for the production of biofuels and other bioproducts (Ragauskas et al., 2014; Beckham et al., 2016; Velvizhi et al., 2022).

A more complete understanding of the molecular processes underlying lignification would benefit future technological developments aiming to deconstruct plant cell walls. Yet, efforts to reduce lignin content and/or alter lignin substructures to alleviate the polymer’s recalcitrant properties often introduce several pleiotropic effects such as stunted plant growth and associated yield penalty (Muro-Villanueva et al., 2019; Vanholme et al., 2019; Ha et al., 2021). Unfortunately, the precise molecular mechanisms underlying the negative effect on plant growth and development in low lignin mutants and transgenics are not well understood. Prevalent hypotheses include restricted nutrient transport through collapsed xylem vessels (Man Ha et al., 2019; Muro-Villanueva et al., 2019), overaccumulation/overproduction of bioactive molecules (Bonawitz and Chapple, 2013; El Houari et al., 2021), deficiency of metabolites critical for normal plant growth (Bonawitz and Chapple, 2013; Muro-Villanueva et al., 2019), and cell wall integrity sensing and stress signaling (Xie et al., 2018; Gallego-Giraldo et al., 2020; Ha et al., 2021) leading to growth defects. Because of the availability of more robust genetic resources, most of these studies have thus far been focused on model dicot plants, such as Arabidopsis and alfalfa, but our knowledge on lignin modification-induced dwarfism in monocotyledonous grass species remains largely unexplored.

Lignin metabolism in grasses differs substantially from that in eudicots (Dixon and Barros, 2019). One difference is that the phenylpropanoid pathway intermediate 4-coumarate is formed entirely from deamination of phenylalanine followed by 4-hydroxylation in dicots, whereas it derives partially from direct deamination of tyrosine in grasses (Barros et al., 2016). In addition, a central lignin gene/enzyme in dicots, caffeoyl-shikimate esterase (CSE), is absent in many grass species (Vanholme et al., 2013; Ha et al., 2016). Recent findings uncovered the existence of a bifunctional peroxidase enzyme, coumarate 3-hydroxylase (C3H/APX), which provides a parallel route to catalyze the direct conversion of 4-coumarate to caffeate in the early steps of lignin biosynthesis in *Brachypodium distachyon* (Barros et al., 2019). C3H/APX is the only soluble non-membrane-bound hydroxylase in the lignin pathway reported to date, and uses molecular oxygen and ascorbate as a reducing agent to oxidize phenolic substrates like 4-coumarate. To characterize the role of C3H/APX, the knockdown of C3H/APX (C3H/APX-KD) was generated by T-DNA insertion in Brachypodium (Hsia et al., 2017). Along with a markedly reduced lignin content, like many other lignin-deficient plants, the C3H/APX-KD exhibited a negative growth phenotype along with higher percentage of non-viable seeds and delayed senescence, providing a suitable biological system to study growth defects associated with lignin depletion in grasses (Barros et al., 2019).

Grass stems contain significantly more lignified cell wall material as compared to leaves and seeds, providing us a rationale to focus the present study in stem tissue (Sarath et al., 2008). Similarly, high-resolution proteomics allows the large-scale identification and quantification of Brachypodium proteins for a system level interrogation of altered biological processes, which will help to connect molecular change to the observed phenotype (Aebersold and Mann, 2016). The objectives of this study were to i) characterize the changes in phenylpropanoid biosynthetic enzymes resulting from knockdown of C3H/APX protein, and ii) gain a deeper understanding of how knockdown of C3H/APX protein leads to negative growth phenotype in the model grass Brachypodium. Therefore, in this study, bottom-up proteomics was utilized to characterize the functional importance of C3H/APX in mature stem tissues of Brachypodium. In support of these measurements, transcriptomics data and metabolomic measurements were obtained to provide additional lines of evidence. From this work, we hypothesize that C3H/APX links the cytosolic route of monolignol precursor hydroxylation to reactive oxygen species metabolism and knockdown of C3H/APX to induce ROS associated growth-defense trade-offs.

## Materials and methods

### Plant material and growth conditions

Brachypodium knockdown lines were obtained from the JGI Brachypodium T-DNA collection (Hsia et al., 2017), and selected as reported previously (Barros et al., 2019). The Brachypodium C3H/APX knockdown line (JJ25124) was identified as an activation tagging silencing mutant transformed with the pJJ2LBA vector and with the T‐DNA inserted into the last intron of the *c3h/apx* gene Bradi1g65820 (I1H6P1). Similarly, Brachypodium APX3 knockdown line (JJ22251) transformed with the same pJJ2LBA vector has the T-DNA inserted near the stop codon of the microsomal ascorbate peroxidase 3 (APX3, Bradi3g42340, I1I9A3) and was used as a T-DNA control. The parental line of the T-DNA mutant population (accession Bd21-3) was used as non-transgenic WT control. Briefly, Brachypodium seeds were sown in the soil, vernalized in the dark at 4°C for 3 days and then moved to a growth chamber with under 70% humidity, and 16-h-light:8-h-dark photoperiod at 24°C, and light intensity of 100 μEm^–2^ s ^–1^. Plantlets were covered with a dome and kept well-watered for 1 week. After this time, plantlets were transferred to half-gallon pots containing Metro-Mix 360 (Sun Gro Horticulture) and grown in the greenhouse for 12 weeks with 70% humidity and 14-h light at 25°C, 10-h dark at 22°C, supplemented with photosynthetically active radiation (PAR) lights when the range was out of 40–120 μEm^–2^ s^−1^ during the day. The growth and lignin phenotypes of C3H/APX-KD Brachypodium lines were assessed and were consistent with the phenotypes of the previously characterized and published C3H mutant in multiple alleles of *A. thaliana* (Barros et al., 2019). Mature stem tissues from 12-week-old plants were collected for the proteomics measurement.

### Proteomics sample preparation

Ground Brachypodium stem tissue samples were suspended in lysis buffer (4% sodium dodecyl sulfate + 10 mM dithiothreitol in 100 mM ammonium bicarbonate (ABC)). Samples were boiled for 5 min at 90°C, sonicated (Branson 450 Digital Sonifier with 30% amplitude, 10 s pulse with 10 s rest, 1 min total pulse time) and boiled for an additional 5 min at 90°C. Samples were centrifuged and supernatants were collected, which were then alkylated by incubating with 30 mM iodoacetamide for 15 min in the dark at room temperature. After alkylating the samples, proteins were then extracted using a chloroform-methanol extraction protocol (Jiang et al., 2004). The extracted protein layer was washed using 100% methanol, air-dried, and reconstituted in 2% sodium deoxycholate (SDC) solution made in 100 mM ABC. Protein concentration was measured using a NanoDrop OneC spectrophotometer (Thermo Scientific). Two hundred and fifty ug of total protein for each sample were digested with two aliquots of sequencing grade trypsin (Promega, 1:75 [wt/wt]) at two different sample dilutions, 2% SDC (3 h) and subsequently 1% SDC (overnight). After digestion, SDC was removed by precipitating with 1% formic acid followed by an ethyl acetate wash. Samples were then lyophilized in a SpeedVac concentrator (Thermo Fischer Scientific). Peptide samples were desalted on Pierce peptide desalting spin columns (Thermo Scientific) as per the manufacturer’s instructions and then resuspended in 0.1% formic acid solution.

### Proteomics LC-MS/MS analysis

Peptides were analyzed by liquid chromatography and tandem mass spectrometry (LC-MS/MS) as previously described (Villalobos Solis et al., 2020) using a Proxeon EASY-nLC 1200 liquid chromatography pump (Thermo Fisher Scientific) coupled to a Q Exactive Plus mass spectrometer (Thermo Fischer Scientific). Briefly, peptides were separated on an in-house-pulled nanospray emitter of 75 μm inner diameter packed with 30 cm of Kinetex C18 resin (1.7 μm, 100 Å, Phenomenex). For each sample, 2 μg aliquots of peptides were loaded in buffer A (0.1% formic acid, 2% acetonitrile) and separated with a linear 210 min organic gradient, followed by a wash and re-equilibration step: 0% to 2% solvent B over 27 min, 2% to 25% solvent B over 148 min, 25% to 50% solvent B over 10 min, 50% to 0% solvent B over 10 min, hold at 0% solvent B for 15 min. The flow rate was kept at 250 nl/min. MS data were acquired with the Thermo Xcalibur software v4.27.19 using the top 10 data-dependent acquisition method. Other spectral data acquisition parameters have been reported before (Villalobos Solis et al., 2020).

### Protein identification and quantification

Raw files were analyzed *via* Proteome Discoverer v2.5 using MS Amanda 2.0 (Dorfer et al., 2014) and confidence in peptide-to-spectrum (PSM) matching was evaluated by Percolator (Kall et al., 2007). Spectral data were searched against the *Brachypodium distachyon* reference proteome database (downloaded on 09/12/2019) from UniProt to which common laboratory contaminants were appended. The following parameters were set up in MS Amanda to derive fully tryptic peptides: MS1 tolerance = 5 ppm; MS2 tolerance = 0.02 Da; missed cleavages = 2; Carbamidomethyl (C, + 57.021 Da) as static modification; and oxidation (M, + 15.995 Da) as dynamic modifications. The percolator false discovery rate (FDR) threshold was set to 1% at the PSM and peptide levels. FDR-controlled peptides were mapped to their respective proteins and protein-level abundance were calculated by summing together peptide extracted ion chromatograms. For quantitative analyses, proteins were required to have at least 1 unique peptide. Protein abundances were LOESS normalized between the biological replicates and mean-centered across the entire dataset on log_2_-transformed values using the InfernoRDN software (Polpitiya et al., 2008). Proteome datasets were further filtered to improve the robustness of the downstream analysis by removing proteins that were not quantified in at least two biological replicates in at least one condition. The missing abundance values on proteins were then imputed with random values drawn from the normal distribution (width 0.3, downshift 2.8) using the Perseus software v.1.6.12.0 (Tyanova et al., 2016). Protein abundances were tested for significant changes across samples by performing an analysis of variance (ANOVA) test followed by Tukey HSD binary comparisons using an in-house written python code using scipy and statsmodels. A protein was considered significantly changing in abundance in C3H/APX-KD line only if it: i) passes a significant threshold of 0.05 in ANOVA, ii) passes a significant threshold of 0.05 in binary comparison of C3H/APX-KD line to WT as well as T-DNA control, and iii) doesn’t pass the significant threshold of 0.05 in binary comparison of WT and T-DNA control.

### Functional enrichment analysis

The differentially abundant proteins data matrix was divided into two groups to contain upregulated and downregulated proteins by hierarchical clustering using the fast Ward’s method. These two groups were used for analyzing enriched gene ontology (GO) terms using the g:Profiler tool (https://biit.cs.ut.ee/gprofiler/gost) (Raudvere et al., 2019). In g:Profiler, *Brachypodium distachyon* was used for the organism, Benjamini-Hochberg FDR of 0.05 was used for significance threshold to correct for multiple hypothesis testing. All other parameters were kept to default. Enriched GO terms were further filtered for redundancy using REVIGO (Supek et al., 2011).

Protein class overrepresentation test was performed using PANTHER v16.0 (Mi et al., 2021). All proteins in the *Brachypodium distachyon* database were used for the background, and a statistical test was done using Fisher’s exact test followed by a PANTHER specific FDR correction with a significance threshold of 0.05.

### Untargeted metabolic profiling

Lyophilized and ground Brachypodium stem samples (~10 mg) were weighed and extracted using 2 ml of 80% ethanol and 50 μl of internal standard (sorbitol 1 mg ml^−1^) as previously described (Barros et al., 2019). One microgram samples were injected into a GC-MS Agilent 5975C inert XL operated in electron impact (EI; 70 eV) ionization mode as described previously (Tschaplinski et al., 2012). Metabolite peaks were extracted using a characteristic mass-to-charge (m/z) ratio to minimize integration of co-eluting metabolites. The extracted peaks were scaled back to the total ion current (TIC) using predetermined scaling factors. Peaks were quantified by area integration and normalized to the quantity of internal standard recovered, amount of sample extracted, derivatized, and injected. A user-created database (>2400 spectra) of EI fragmentation patterns of TMS-derivatized compounds and the Wiley Registry 10^th^ Edition/NIST 2014 Mass Spectral Library were used to identify the metabolites in the samples. Unidentified metabolites were designated by their retention time and key m/z ratios.

### RNA-seq data analysis

Brachypodium and Arabidopsis C3H/APX-KD and WT stem samples were used for the RNA-seq analysis. Fragments Per Kilobase of transcript per Million mapped reads (FPKM) output data was processed using the tag count comparison (TCC) R package through TCC-Graphical User Interface (TCC-GUI v.2021.11.13) (Su et al., 2019). The Trimmed Mean of M-values (TMM) normalization method with the Bioconductor package edgeR were used to filter differentially expressed genes (DEGs) (Su et al., 2019). Genes with count lower than 15 FPKM were filtered. A p-value < 0.05 and log_2_FC of greater than 1 or less than -1 were considered significant. The log_2_FC values were based on “M Value” column (provides log-ratios = log_2_ (Group#2/Group#1) from TCC-GUI. Genes differentially expressed were analyzed for Gene Ontology (GO) enrichment for biological process using AgriGO under Poaceae group (Du et al., 2010; Tian et al., 2017). *Brachypodium distachyon* (Phytozome v11.0) was used for the reference database and all other parameters were set at default (Du et al., 2010; Tian et al., 2017).

### Reactive oxygen species (ROS) and H_2_O_2_ measurement

Brachypodium mutant and control plants were germinated and grown for 4-weeks in Jiffy peat pellets under controlled conditions of 25°C and 10-h light/14-h dark cycle, 50 μmol photons s^−1^ m^−2^. To impose oxidative stress, plants were fumigated for 30 min with 1 μM paraquat and 50 μM H_2_DCFDA in 50 mM phosphate buffer (pH 7.4) and 0.001% Silwet L-77 using a nebulizer in a glass container as previously described (Fichman et al., 2019). After fumigation, plants were exposed to high light stress (1,100 μmol photons s^−1^ m^−1^) using an array of LED lights (BESTVA) for 30 min. Plants were immediately placed in a fluorescence imager (IVIS Lumina S5) and imaged for ROS detection (excitation/emission: 480 nm/520 nm). The analysis of the images was conducted using the Living Image software (PerkinElmer). Following the imaging, the aerial parts of the plants were fast-frozen in liquid nitrogen and ground to a fine powder for H_2_O_2_ measurement using Amplex Red (Fichman et al., 2022). Briefly, two hundred microliters of 0.1% trichloroacetic acid was added to ground tissues and samples were centrifuged at 12,000 g for 12 min at 4°C. Following the centrifugation, 100 μl of supernatant was mixed with 400 μl of 100 mM sodium phosphate buffer (pH 7.4) for pH balance. One hundred microliters of supernatant were transferred into fresh tubes and incubated with 100 μl of phosphate buffer containing 50 μM Amplex Red and 0.05 U ml^−1^ horseradish peroxidase for 30 min at room temperature in the dark. The concentration of H_2_O_2_ in each sample was quantified by fluorescence spectrophotometry using a Qubit Fluorometer (Invitrogen) from a standard curve from 0-25 μM of H_2_O_2_. Following the measurement, tissue samples were completely dried using a speed vacuum concentrator at 60°C for 2h min and H_2_O_2_ accumulation per mg dry weight was calculated.

## Results

### C3H/APX knockdown leads to substantial alterations in the Brachypodium stem proteome

**Figure 1:**
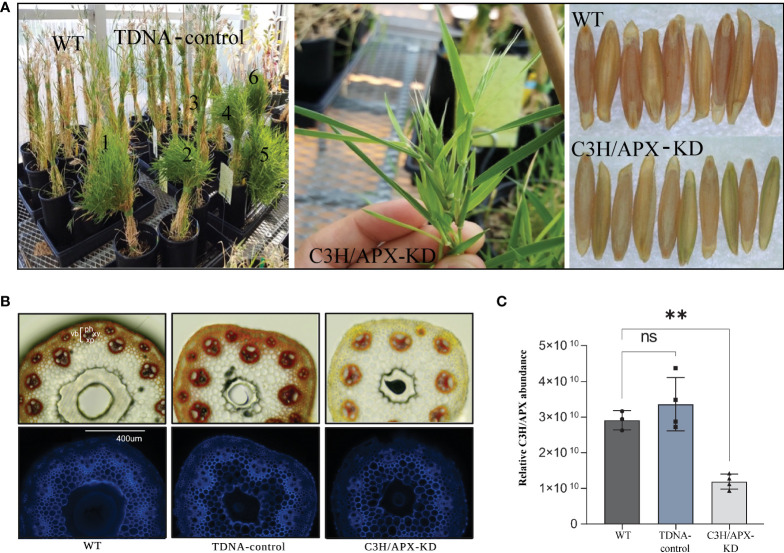
Phenotypic characterization and Brachypodium C3H/APX protein abundance profile across experimental conditions. **(A)** C3H/APX-KD lines (numbered 1-6 in panel 1) exhibit stunted growth, delayed leaf senescence (stay-green phenotype) and aborted seeds. **(B)** Transverse sections (phloroglucinol-HCl staining and UV autofluorescence) of Brachypodium stems from wild-type (WT), -DNA controls and C3H/APX-KD lines. In the phloroglucinol-HCL image, G-lignin is red stained; S lignin is peach orange stained. ‘ph’ represents phloem; ‘xv’ represents xylem vessels; ‘xp’ represents xylem parenchyma and ‘vb’ represents vascular bundle. UV autofluorescence images were captured with an EVOS FL Cell Imaging System (Thermo Fisher) equipped with a DAPI led light cube (excitation: 357/44 nm; emission: 447/60 nm). Overall, C3H/APX-KD does not appear to have collapsed vessel walls. **(C)** Bar chart showing the targeted knockdown of C3H/APX (I1H6P1) resulted in substantially lower protein abundance (~60% lower abundance when compared to WT). ‘ns’ represent not significant. ** represent significant differences (adj p-value < 0.01) among genotypes.

To better understand the molecular implications of the C3H/APX knockdown (**Figures 1A, B**), we analyzed the Brachypodium C3H/APX-KD, T-DNA controls, and wild-type controls (WT) using comparative proteomics. Proteomics measurement identified C3H/APX protein with 56% coverage (**Supplement Figure 1A**) and as expected, the C3H/APX protein showed ~60% and 65% lower abundance in the Brachypodium C3H/APX-KD when compared to the WT and TDNA controls, respectively (**Figure 1C**). Besides the decrease in C3H/APX protein abundance, Brachypodium C3H/APX-KD also showed a significant reduction of microsomal APX (mAPX), stromal APX (sAPX), and thylakoid membrane bound APX (tAPX) protein abundances (**Supplement Figure 1B**).

**Figure 2:**
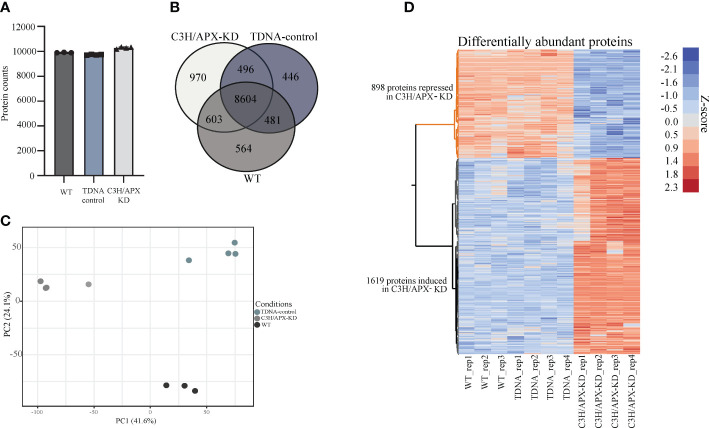
Global assessment of proteomics measurements. **(A)** The number of proteins identified across experimental conditions. The number of proteins identified ranges from around 9,600 to 10,400. Each experimental condition has at least biological triplicates. **(B)** Venn diagram of the identified proteins in each experimental condition. Majority of the proteins were identified in all conditions with ~ 4-8% uniquely identified in individual treatment condition. **(C)** Principal component analysis (PCA) for the experimental samples. PCA of proteome data showed discrete grouping between the different experimental conditions. **(D)** Heatmap of proteins that are significantly changing in abundance between controls (WT and TDNA control) and the C3H/APX-KD.

At a 1% false discovery rate, 12,164 proteins were identified across genotypes, with the highest number (~10,300) of proteins being observed in the C3H/APX-KD, followed by the WT (~9,900) and T-DNA control (~9,700) (**Figure 2A** and **Supplement Table 1**). Approximately 70% of the proteins identified were present in all sampling conditions with 4.6%, 3.7%, and 8.0% proteins identified uniquely in C3H/APX-KD lines, WT, and T-DNA controls, respectively (**Figure 2B**). Principal component analysis (PCA) of proteome data showed discrete grouping between the different treatment lines (**Figure 2C**). To identify changes in protein abundance resulting due to knockdown of C3H/APX, ANOVA followed by Tukey HSD binary comparisons was performed. In total, 2,517 proteins (21.2% of the total identified proteome) were significantly different in abundance when comparing C3H/APX-KD lines to both controls. Among the proteins with altered abundances in C3H/APX-KD lines, 898 proteins had significantly lower abundances, and 1,619 proteins had significantly higher abundances in C3H/APX-KD when compared to both controls (**Figure 2D** and **Supplement Table 1**).

### C3H/APX deficiency impacts enzymes associated with the phenylpropanoid biosynthesis pathway

**Figure 3:**
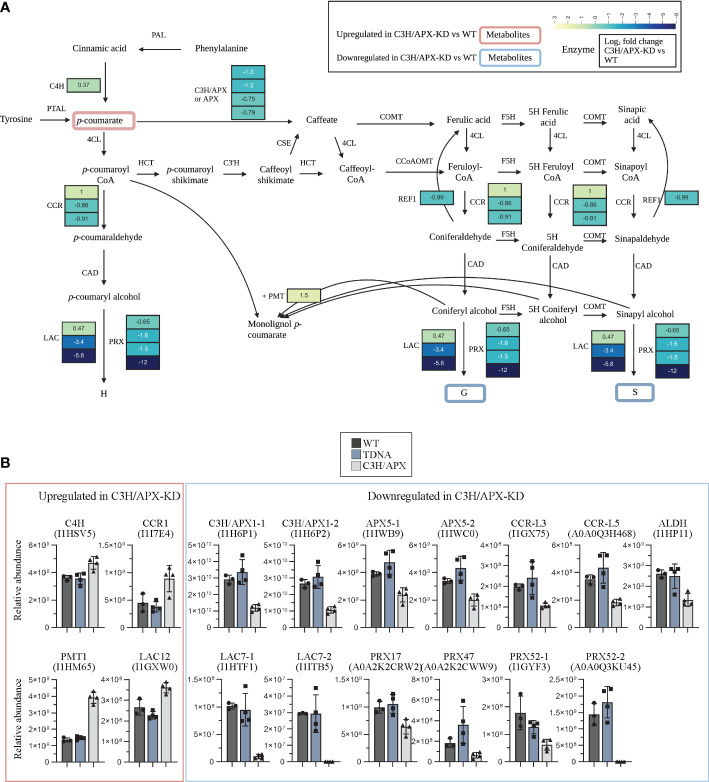
Impact of C3H/APX knockdown in lignin biosynthesis pathway. **(A)** scheme of the lignin biosynthesis pathway is overlaid with significantly changing proteins and metabolites in C3H/APX-KD compared to WT. The heat blocks represent the log_2_ fold change obtain from the binary comparison of C3H/APX-KD with WT. All proteins showed passed significance threshold. CCR isoforms shown are I1I7E4 (CCR1), I1GX75 (CCR-like3) and A0A0Q3H468 (CCR-like5). LAC isoforms shown are I1GXW0 (LAC12), I1HTF1 (LAC7-1) and I1ITB5 (LAC7-2). PRX isoforms shown are A0A2K2CRW2 (PRX17), A0A2K2CWW9 (PRX47), I1GYF3 (PRX52-1) and A0A0Q3KU45 (PRX52-2). Detailed information about these proteins is provided in Supplemental Table 2. The red or blue outlines in metabolites represent significantly up or down-regulated metabolites in C3H/APX-KD compared to WT. **(B)** Bar plots below the pathway show relative abundance of the significantly changing proteins in all experimental conditions. A flavonoid pathway marker, CHS were significant but are not shown in this pathway. PAL, L-phenylalanine ammonia-lyase; C4H, cinnamate 4-hydroxylase; COMT, caffeate/5-hydroxyferulate 3-*O*-methyltransferase; F5H, ferulate 5-hydroxylase/coniferaldehyde 5-hydroxylase; 4CL, 4-hydroxycinnamate; CoA ligase, HCT, 4-hydroxycinnamoyl; CoA, shikimate/quinate hydroxycinnamoyltransferase; C3’H, 4-coumaroyl shikimate/quinate 3’-hydroxylase; CSE, caffeoyl shikimate esterase; CCoAOMT, caffeoyl CoA 3-*O*-methyltransferase; CCR, cinnamoyl CoA reductase; CAD, cinnamyl alcohol dehydrogenase; PMT, *p-*coumarate monolignol transferase; LAC, laccase; PRX, Peroxidase; REF, reduced epidermal fluorescence; PTAL, bifunctional L-phenylalanine/L-tyrosine ammonia-lyase; C3H/APX, 4-coumarate 3-hydroxylase; APX, Ascorbate peroxidase; CHS, chalcone synthase.

As a bifunctional enzyme associated with phenylpropanoid metabolism, it remains to be determined how reduced C3H/APX function impacts the abundance of lignin biosynthesis-related proteins. Of the 87 potential candidate lignin biosynthetic proteins in Brachypodium (Barros et al., 2022), 62 were identified in the current study, and 14 were significantly altered in protein abundances as per our criteria (**Supplement Table 2**). The proteomics analysis showed that the reduction of C3H/APX abundance in stem tissues led to increased levels of cinnamoyl-CoA reductase (CCR; I1I7E4; Bradi3g36887), which catalyzes the NADPH-dependent conversions of *p*-coumaroyl-CoA, feruloyl-CoA, and sinapoyl-CoA into the corresponding aldehydes. Similarly, *p*-coumarate monolignol transferase (PMT; I1HM65; Bradi2g36910), a grass-specific acyltransferase enzyme that acylates monolignols with *p*-coumarate, also increased in abundance when compared to controls. Besides CCR and PMT, a small increase in abundance of cinnamic acid 4-hydroxylase (C4H1; I1HSV5; Bradi2G53470) and laccase-12 (LAC12; I1GXW0; Bradi1G37620) was observed in C3H/APX-KD as compared to controls. Apart from these, we observed 10 proteins with significantly lower abundances in C3H/APX-KD compared to controls. Among these reduced-abundance proteins were two laccase-7 proteins (I1HTF1; Bradi2G55050 and I1ITB5; Bradi4G39330), two CCR-like proteins (I1GX75; Bradi1g35730 and A0A0Q3H468; Bradi1g35736), two C3H/APX proteins (I1H6P1 & I1H6P2; Bradi1g65820), two ascorbate peroxidases APX5 (I1IWB9; Bradi5g03640 and I1IWC0; Bradi3g42340), aldehyde dehydrogenase (ALDH) known as reduced epidermal fluorescence REF1 (I1HP11; BRADI2g42360) and the early flavonoid pathway enzyme chalcone synthase (I1ILF3; Bradi4g17230) (**Figure 3**). We also profiled the protein abundance of other peroxidases previously reported to be implicated in lignin polymerization (Tokunaga et al., 2009; Fernandez-Perez et al., 2015; Cosio et al., 2017; Hoffmann et al., 2020) including PRX17 (Cosio et al., 2017; Hoffmann et al., 2020), PRX47 (Tokunaga et al., 2009) and PRX52 (Fernandez-Perez et al., 2015) all with decreased abundance in C3H/APX-KD compared to controls (**Figure 3** and **Supplement Table 2**).

Given that phenylpropanoid metabolism is tightly linked to the shikimate pathway, with shikimate acting as an upstream precursor of 4-coumaroyl-CoA as well as a substrate of hydroxycinnamoyl CoA: shikimate hydroxycinnamoyl transferase/coumaroyl shikimate 3-hydroxylase (HCT/C3′H) mediated 3-hydroxylation (Hoffmann et al., 2004; Qian et al., 2019), we extended the focus onto how the enzymes of the shikimate pathway may be changing in the C3H/APX-KD line. Interestingly, the proteomics analysis identified the majority of the proteins involved in shikimate and aromatic amino acid biosynthesis pathways, with several proteins such as shikimate kinase, arogenate dehydratase, and arogenate dehydrogenase being substantially upregulated in C3H/APX-KD when compared to both controls (**Supplement Figure 2** **)**. We then shifted the focus to the flavonoid biosynthetic pathway which is also linked to the lignin biosynthesis pathway. Proteomics analysis showed that early flavonoid biosynthetic proteins like naringenin-chalcone synthase and flavanone 3-dioxygenase were significantly downregulated, whereas late flavonoid biosynthetic proteins such as anthocyanidin 3-*O*-glucosyltransferase were significantly upregulated in C3H/APX-KD compared to controls (**Supplement Table 2**).

### C3H/APX knockdown altered abundance of transcriptional regulators involved in the phenylpropanoid biosynthesis pathway

**Figure 4:**
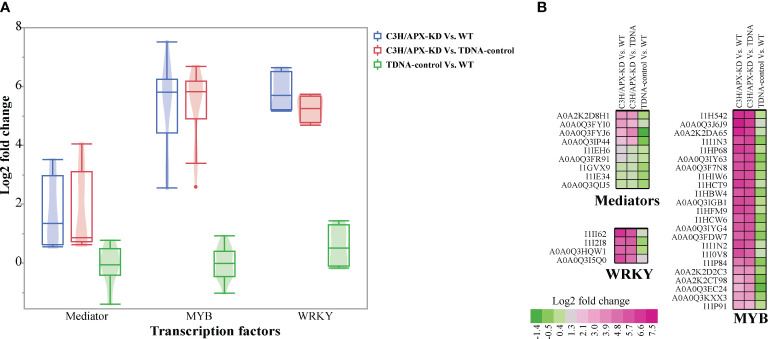
Protein abundance profile of transcriptional regulators. **(A)** Boxplot shows the overall abundance change of three type of transcription factors. The violin plot inside the boxplot shows the distribution of data. **(B)** Cell plot shows the log2 fold change for mediators, MYB, WRKY. All the proteins represented passes significant threshold (adj p-value < 0.05). Color gradients represent the log_2_ fold change.

Transcription factors tightly modulate the phenylpropanoid biosynthesis pathway (Zhao and Dixon, 2011; Bonawitz et al., 2012; Bonawitz et al., 2014; Muro-Villanueva et al., 2019; Zhang et al., 2020). To assess how the knockdown of an enzyme involved in the phenylpropanoid biosynthesis pathway affects transcriptional regulation, we profiled the abundance of transcriptional factors across experimental conditions. Overall, we observed an increased abundance of transcriptional regulators such as Mediator complex, MYB, and WRKY (**Figure 4A** and **Supplement Table 3**). Proteomics analysis identified nine significantly changing subunits of the Mediator complex (including MED9, MED26C, MED23, and MED36B). All Mediator proteins identified were upregulated in the C3H/APX-KD line compared to controls, with 6 of them >1 log_2_ fold higher in abundance than in both controls (**Figure 4B** and **Supplement Table 3**). Similarly, our analysis showed that 23 MYB transcription factors were substantially upregulated, with at least a 2.5 log_2_ fold change in the C3H/APX-KD line compared to both controls (**Figure 4B** and **Supplement Table 3**). A recent study identified an importin-beta-like protein that mediates the transport of the MYB4 transcriptional repressor from the cytosol into the nucleus (Zhao et al., 2007; Panda et al., 2020). In Arabidopsis, importin-beta-like protein and MYB4 have been shown to mediate lignin modification-induced dwarfism, and suppression of these genes alleviates the dwarf phenotype (Panda et al., 2020). Interrogation of Brachypodium orthologs revealed 3 importin-beta-like proteins upregulated by at least 1 log_2_ fold in C3H/APX-KD when compared to controls (**Supplement Table 3**). Likewise, MYB4 (A0A0Q3J6J9; Bradi2g40620) was 7.5 log_2_ fold higher in abundance in C3H/APX-KD when compared to controls. In addition to these, 4 WRKY transcription factors were substantially upregulated with at least 5.2 log_2_ fold in the C3H/APX-KD line when compared to controls (**Figure 4B**).

### C3H/APX suppression alters the abundance of the proteins associated with phytohormone signaling and senescence

Brachypodium C3H/APX-KD lines exhibit a dwarf and stay green (delayed senescence) phenotype (**Figure 1A**). Phytohormones have been known to impact the progression and regulation of the senescence process (Joshi et al., 2019; Guo et al., 2021). Additionally, alteration in phytohormones can also result in reduced-growth and dwarf phenotypes (Fujioka and Yokota, 2003; Thomas and Sun, 2004) and previous studies indicate modifying lignin can alter phytohormone levels (Besseau et al., 2007; Gallego-Giraldo et al., 2011). To understand how phytohormones are regulated in C3H/APX-KD Brachypodium, proteins associated with phytohormone biosynthesis and perception were interrogated. Overall, significantly changing abundances were observed for proteins related to all eight phytohormones (**Supplement Figure 3A** and **Supplement Table 4**). Among these, proteins associated with abscisic acid (ABA) and auxin were the most represented and showed increased abundances in the C3H/APX-KD line (**Supplement Figure 3A** and **Supplement Table 4**).

For ABA, we observed a significant increase in ABA regulating proteins such as ABA receptor PYL8, ABA-regulated RNA-binding protein (APR1), THO complex subunit 6 (THOC6) in the C3H/APX-KD line compared to both controls (**Supplement Table 4**). Meanwhile, a significant decrease in ABA biosynthesis proteins such as Zeaxanthin epoxidase (ZEP), HVA22-like protein, and serine/threonine-protein kinase SRK2 were observed in the C3H/APX-KD line compared to controls (**Supplement Table 4**). SRK2 plays a key role in abscisic acid (ABA) signaling and helps to regulate seed germination and seedling growth (Kim et al., 2012). It interacts with bZIP transcription factors in rice which were also found to be reduced in C3H/APX-KD by 7.17 log_2_ fold of WT.

For auxin, our analysis identified substantial decreases in proteins involved in auxin response such as auxin-induced in root cultures (AIR) protein in the C3H/APX-KD line compared to both controls (**Supplement Table 4**). The WAT1 related protein (A0A2K2DKI4; Bradi1g21860) was one of the most significantly decreased proteins (~ 9.94 log_2_ fold lower than WT) in the C3H/APX-KD line (**Supplement Table 4**). WAT1 is a transmembrane protein that is involved in growth and secondary cell wall development in Arabidopsis (Ranocha et al., 2010; Ranocha et al., 2013). It has also been reported to be involved in auxin transport and homeostasis (Ranocha et al., 2013) and may suggest why the Brachypodium C3H/APX-KD line has reduced growth and development. Proteins negatively regulated by auxins such as dormancy-associated protein homologs (DRMHs) were substantially increased in abundance (**Supplement Table 4**) (Rae et al., 2013).

Besides phytohormones, the senescence process is also highly coordinated through senescence-associated genes (SAGs). Proteomics analysis identified two significantly changing SAGs which were both substantially downregulated in C3H/APX-KD compared to controls (**Supplement Figure 3B**). Meanwhile, senescence-associated e3 ubiquitin ligase 1 (I1IFT9; Bradi3g60810) which prevents premature senescence was significantly upregulated in C3H/APX-KD compared to controls (**Supplement Figure 3B**).

### C3H/APX deficient Brachypodium exhibit disrupted redox homeostasis, photosynthesis, and DNA repair

C3H/APX encodes a cytosolic ascorbate peroxidase and therefore it is anticipated that its reduced expression will impact biological processes influenced by redox homeostasis (Agrawal et al., 2003; Davletova et al., 2005). For the proteins having reduced abundance in C3H/APX-KD lines, a gene ontology (GO) enrichment test showed a significant overrepresentation of GO terms related to ROS metabolic processes, cellular oxidant detoxification and hydrogen peroxide catabolic processes (**Supplement Figure 4A**; **Supplement Table 5**). Among proteins related to oxidative stress responses, peroxidase, sulfiredoxin, ABC1-like kinase, class I heat shock protein, and peroxiredoxin were substantially reduced in abundance in C3H/APX-KD compared to controls (**Supplement Table 5**).

**Figure 5:**
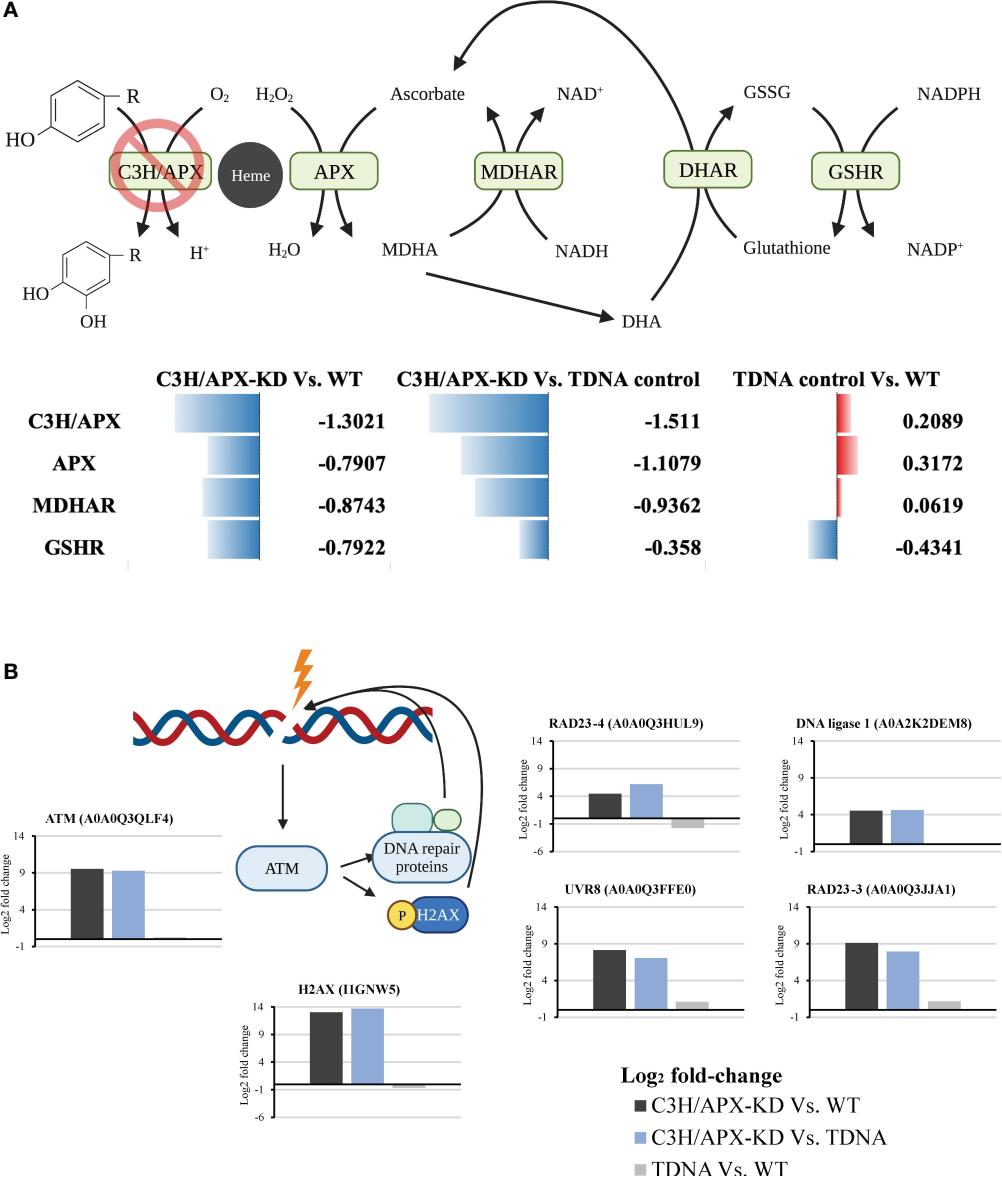
C3H/APX-deficient Brachypodium exhibit altered redox homeostasis and DNA damage response. **(A)** Schematic overview of ascorbate recycling pathway involved in plant redox homeostasis. Multiple enzymes involved in ascorbate recycling were significantly decreased in abundance in C3H/APX-KD compared to controls. Numbers next to data bars are the log_2_ fold change from binary comparison. DHAR was identified but did not pass significant threshold (adj p-value<0.05). APX: L-ascorbate peroxidase; MDHAR: monodehydroascorbate reductase; DHAR: dehydroascorbate reductase; GSHR: glutathione reductase. **(B)** Simplified schematic diagram showing the plant DNA damage response upon cellular stress overlaid with bar plot showing the log_2_ fold changes in each binary comparison for individual proteins involve in the process. All the proteins represented passed the significance threshold of adj p-value<0.05 and number of replicates used were at least 3. ATM, Ataxia telangiectasia mutated; H2AX, Histone H2A; RAD, DNA repair protein; UVR, ultraviolet-B receptor.

Cytosolic ascorbate peroxidase is also involved in the recycling of ascorbate (Asc), the most abundant water-soluble antioxidant, *via* the ascorbate-glutathione cycle (**Figure 5A**). Through this cycle, cellular Asc content and redox state are maintained, which is critical under conditions with increased reactive oxygen species (ROS) production (Gallie, 2013). When compared to controls, proteome expression changes in the C3H/APX-KD line showed a significant reduction in protein abundances associated with ascorbate recycling including monodehydroascorbate reductase (MDHAR) and glutathione reductase (GSHR) (**Figure 5A**).

Previous studies have demonstrated that reduced cytosolic ascorbate peroxidase function leads to disrupted oxidative protection of chloroplasts that results in lower photosynthetic rates and slower growth under normal growth conditions (Pnueli et al., 2003; Miller et al., 2007; Bonifacio et al., 2011; Ribeiro et al., 2012). Corroborating previous studies, our proteome-wide functional analysis showed that biological processes related to photosynthesis, including light harvesting and light reaction functions, were significantly enriched among the proteins repressed in the C3H/APX-KD lines when compared to controls (**Supplement Figure 4** and **Supplement Table 5**). In particular, the relative abundances of chloroplast proteins such as NAD(P)H-quinone oxidoreductase, serine/threonine-protein kinase STN8, and chlorophyll a-b binding protein were significantly lower in the C3H/APX-KD line compared to controls (**Supplement Table 5**).

Among the proteins with significantly increased abundances in the Brachypodium C3H/APX-KD line when compared to controls, the PANTHER protein class overrepresentation test identified damaged DNA-binding proteins to be overrepresented (**Supplement Figure 4B**). Specifically, substantial upregulation of a protein ataxia-telangiectasia mutated (ATM) homolog (A0A0Q3QLF4; Bradi2g00610, log_2_ fold >9.56) was identified in the C3H/APX-KD line compared to controls (**Figure 5B**). ATM is involved in regulating DNA damage responses; it phosphorylates the histone variant H2AX at double-strand breaks (Bakkenist and Kastan, 2003; Podhorecka et al., 2010). Although the phosphorylation profile was not measured in this study, H2AX (I1GNW5; Bradi1g10390) was the most abundant differential protein between the C3H/APX-KD line and WT with a log_2_ fold change of 13.0 (**Figure 5B**). ATM is also involved in regulating other proteins involved in DNA damage responses, such as the DNA repair protein RAD, RCC1 domain-containing protein, and DNA ligase 1, which were all significantly upregulated in C3H/APX-KD compared to controls (**Figure 5B**). Moreover, we also observed the concomitant upregulation of organelle DNA repair proteins (I1HS48; Bradi2g51390 and A0A0Q3JCJ2; Bradi2g51390) that repair organelle-specific DNA double-stranded breaks by microhomology-mediated end-joining (Garcia-Medel et al., 2019).

### C3H/APX deficiency impacts cellular kinases implicated in plant defense

Non-receptor serine/threonine kinases are intracellular cytoplasmic proteins that relay intracellular signals (Siveen et al., 2018) and were found to be differentially regulated in the C3H/APX-KD line compared to controls (**Supplement Figure 4B**). Significantly changing proteins mapping to this protein class were extracted and qualitatively analyzed based on ranking fold changes between the C3H/APX-KD line and WT (**Supplement Table 5**). In general, most upregulated non-receptor kinases are implicated in defense signaling such as EDR (Hiruma et al., 2011), and cellular development and expansion like M3KE1 (Chaiwongsar et al., 2012) or NEK5 (Sakai et al., 2008) (**Supplement Table 5**). On the other hand, down regulated non-receptor kinases have been associated with regulation of stress tolerance such as SRK2 (Umezawa et al., 2004), phosphorylation of acidic proteins such as casein, calcium signal transducer like CDPK3 (Mori et al., 2006) (**Supplement Table 5**).

**Figure 6:**
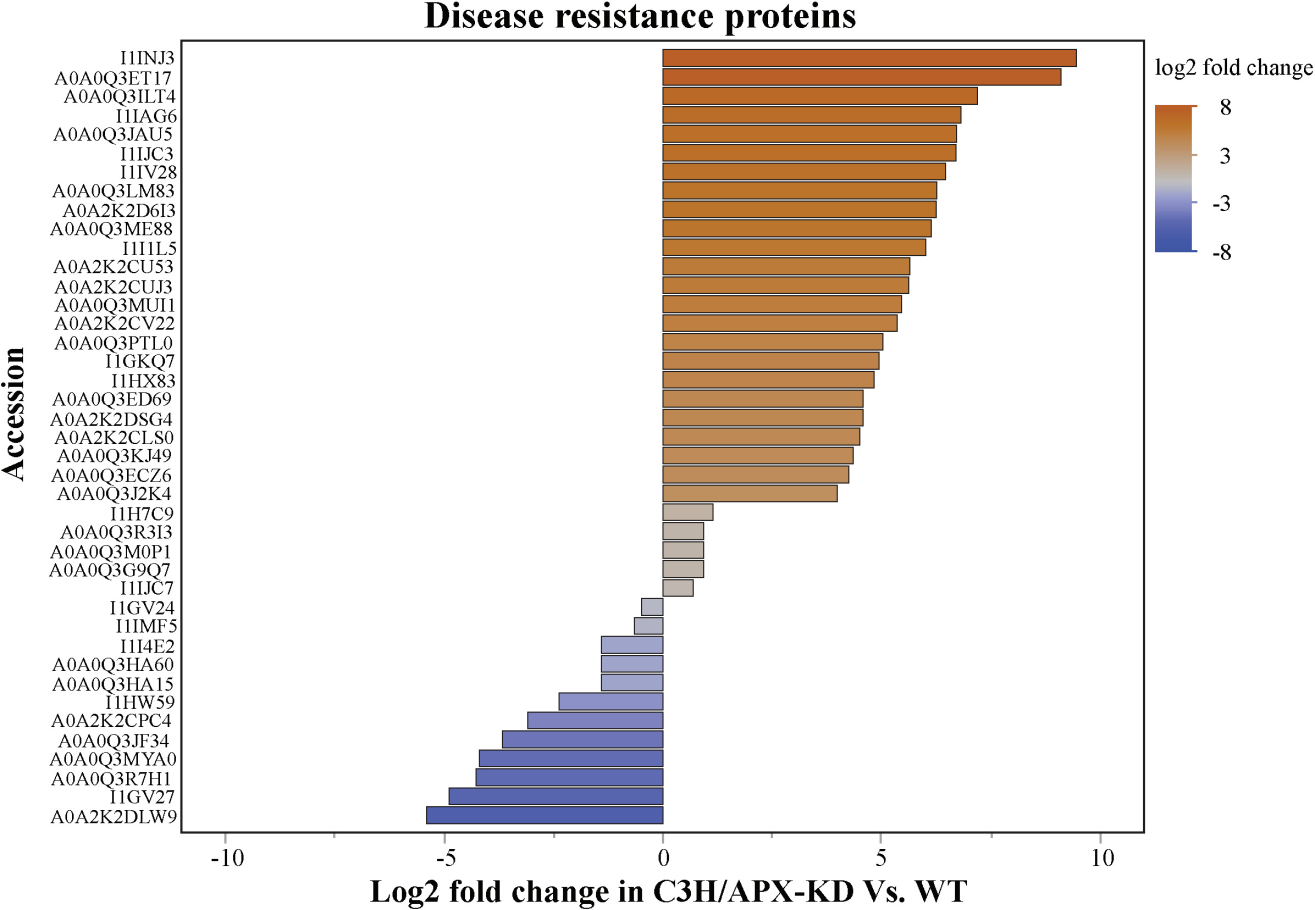
C3H/APX-deficient Brachypodium exhibit elevated levels of disease resistance proteins. Bar plot showing the log_2_ fold change of disease resistance protein in C3H/APX-KD compared to WT. All the protein represented passes the ANOVA significance followed by Tukey HSD binary comparisons significance (adj p<0.05). Blue bars represent the significantly downregulated proteins in C3H/APX-KD compared to controls and orange bars represent the significantly upregulated proteins in C3H/APX-KD compared to controls. Color gradients represent the log_2_ fold gradient. Further information about these proteins are provided in **Supplement Table 5**.

Several studies have shown that defense responses frequently come at the cost of reduced growth in plants (Stamp, 2003; Huot et al., 2014; Karasov et al., 2017; Ha et al., 2021). Because the C3H/APX-KD line displayed abnormal growth phenotype and the proteome-wide analysis shows defense signaling kinases to be differentially regulated, proteins that have been associated with disease resistance were interrogated qualitatively using keywords. Overall, several proteins associated with disease resistance (25 proteins with log_2_ fold>1 vs 10 proteins with log_2_ fold < -1) were differentially abundant between the C3H/APX-KD line when compared to controls (**Figure 6** and **Supplement Table 5** **)**. In addition, pathogen-associated molecular patterns-induced protein (A0A0Q3K2Z8; Bradi2g18237) was increased by 3.56 log_2_ fold in the C3H/AP-KD compared to WT.

### Transcriptomics, metabolomics, and ROS levels support proteomics findings

To obtain information on the transcript level changes in C3H/APX-KD, RNA-seq was performed on C3H/APX-KD and WT Brachypodium. Transcript level analysis identified 470 genes that were differentially expressed. Among these DEGs, 293 genes were significantly upregulated, and 177 genes were significantly downregulated in C3H/APX-KD compared to WT (**Supplement Figure 5A**). When comparing DEGs to significantly changing proteins, we identified 54 significantly changing proteins whose transcript levels were also differentially expressed. Correlation analyses showed a positive correlation (R^2^ = 0.61) between transcript and protein levels (**Supplement Figure 5B**). Overall, the DEGs were enriched in biological processes associated with regulation of transcription and cellular responses to stress (**Supplement Figure 5C**). At the individual gene level, we observed the increased expression of pathogen-associated molecular patterns-induced gene (Bradi2g18237, log_2_FC=2.5) in C3H/APX-KD compared to WT, comparable to the results observed at the proteome levels. Similarly, we found decreased expression levels of the lignin biosynthetic gene CCR2 (Bradi1g35736, log_2_FC= -1.3), senescence related gene SAG12 (Bradi4g07130, log_2_FC= -1.3), and peroxidases such as peroxidase 12 (Bradi5g27150, log_2_FC= -2.6) and peroxidase 52 (Bradi1g41115, log_2_FC= -1.7) (**Supplement Table 6**). Like Brachypodium, Arabidopsis *c3h/apx* mutants exhibited similar molecular changes with increased expression of genes related to oxidative response, defense response and decreased expression of gene related to phytohormone response (**Supplement Table 6**). In general, the transcriptome-level characterization of the C3H/APX-KD line was correlated and consistent with the proteome findings.

Metabolomics was performed to identify changes in metabolites as a result of C3H/APX downregulation. Overall, 91 metabolites were identified, of which 12 metabolites were uniquely upregulated and 8 metabolites were uniquely downregulated in C3H/APX-KD compared to control plants (**Supplement Table 7**). Metabolomics measurement showed significant upregulation of *p-*coumarate, the substrate of C3H/APX, and downregulated levels of G-lignin and syringyl lignan glycoside in C3H/APX-KDs compared to WT and T-DNA controls (**Figure 3** and **Supplement Table 7**). The increased levels of C4H protein and 4-coumarate (product of the C4H reaction) in the C3H/APX-KD lines suggest that at least part of the 4-coumarate levels may derive from higher C4H activity rather than from only downregulation of the target C3H reaction. In addition to metabolites specific to lignin biosynthesis, compounds involved in ROS signaling and abiotic stress response such as linoleic acid and aconitic acid were significantly upregulated in the C3H/APX-KD lines (**Supplement Table 7**). In contrast, the levels of a conjugate of ascorbic acid and gentisic acid-5-*O*-glucoside potentially involved in alleviating oxidative stress in plants were substantially decreased in C3H/APX-KD compared to WT (**Supplement Table 7**).

**Figure 7:**
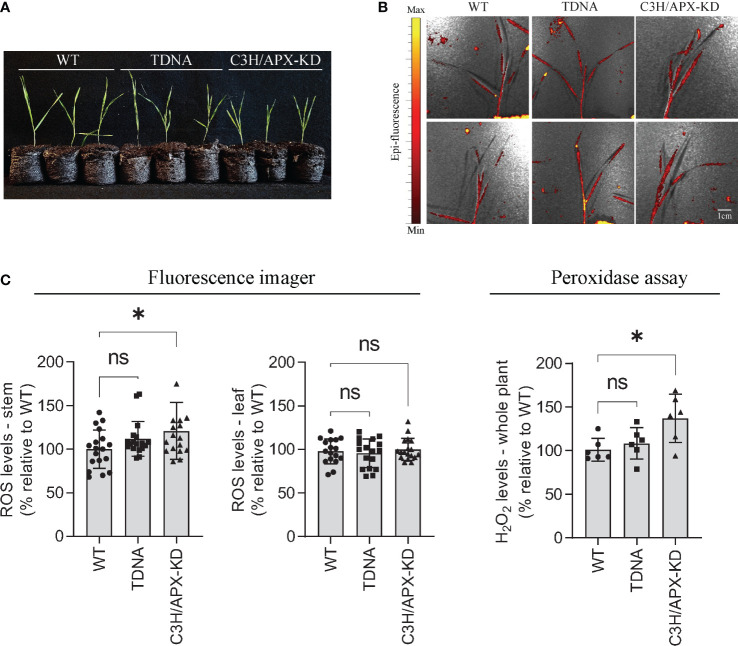
Reactive oxygen species (ROS) and H_2_O_2_ levels in Brachypodium C3H/APX-KD lines. **(A)** C3H/APX-KD lines and controls grown in Jiffy peat pellets for 4-weeks. **(B)** Imaging of ROS in whole plants fumigated with paraquat and stressed under high-light conditions for 30 min each. **(C)** Measurements of ROS levels in the stem and leaf tissues and total H_2_O_2_ accumulation in Brachypodium C3H/APX-KD lines and control plants. Student’s t-test, error bar indicates SD, *P < 0.05, n > 6. Scale bar represents 1 cm. ‘ns’ represent not significant.

To validate a potential misregulation of the redox state supported by the multiple omic datasets, we measured the levels of ROS and hydrogen peroxide in C3H/APX-KD and control plants (**Figure 7**). To avoid free radical damage in the stored plant material, plants were grown again under controlled conditions for 4-weeks (**Figure 7A**), fumigated with the herbicide paraquat and exposed to high-light to induce oxidative stress, and immediately tested for ROS and H_2_O_2_ accumulation. Fluorescence imaging detected higher levels of ROS in the stems of C3H/APX-KDs compared to controls (**Figures 7B, C**). Moreover, the total concentration of H_2_O_2_ in whole C3H/APX-KDs plants detected by fluorescence spectrophotometry was significantly elevated when compared to both TDNA and WT controls (29% and 37% increase, respectively) (**Figure 7C**). ROS and H_2_O_2_ measurements were limited to 4-weeks old plants and we anticipate that a longer growth period would better separate out differences between lines.

## Discussion

Although lignin provides essential mechanical support for plant cell walls, it is a major contributor to biomass recalcitrance of biofuel crops (Boerjan et al., 2003; Velvizhi et al., 2022). Thus, there has been a special interest in modifying the content and/or composition of this important plant biopolymer to facilitate bioprocessing. However, attempts to modify lignin result in pleiotropic effects with several unfavorable traits, including reduced growth. Despite several hypotheses, the precise molecular mechanisms underlying the negative growth of lignin-modified plant remains largely unanswered. Moreover, most of the studies have been primarily focused on dicot plants, and lignin metabolism in grasses, which differs substantially from that in dicots, is understudied.

Recent research in grass species has identified a monolignol pathway enzyme, C3H/APX, which provides a parallel route to the shikimate shunt pathway (Barros et al., 2019). As in many other lignin-modified plants, knockdown of C3H/APX reduces plants lignin content but results in negative growth phenotypes (Barros et al., 2019). In lignin knockdown lines, collapse of xylem vessels which impair water and solute conductance has been hypothesized as one of the likely causes of perturbed growth; however, C3H/APX-KD knockdown plants do not exhibit abnormal vessel walls (**Figure 1**). The precise molecular alterations underlying the negative growth effect observed due to C3H/APX knockdown remain to be explored. In this study, we performed a comparative proteomics analysis in C3H/APX-KD, TDNA control and WT Brachypodium stem tissue to investigate underlying mechanism of C3H/APX-KD associated negative growth and gain a deeper understanding of how grasses alter levels of lignin pathway enzymes and other biological processes following C3H/APX knockdown.

Monolignols are synthesized in the cytoplasm and transported to the cell wall, where they are oxidized by laccases and/or peroxidases prior to their incorporation into the polymer (Bonawitz and Chapple, 2010; Dixon and Barros, 2019). In Arabidopsis, mutation of laccase proteins has been shown to reduce the lignin content (Liang et al., 2006; Zhao et al., 2013). The substantial decrease in abundance of laccase proteins observed from proteomics measurement in C3H/APX-KD compared to controls suggests that the C3H/APX-KD may have reduced ability to oxidize monolignols, which might contribute to their low total lignin. Besides lignin quantity, lignin composition can also be controlled at the polymerization level (Tobimatsu and Schuetz, 2019; Zhuo et al., 2022). Our proteomics and transcriptomics analyses identified several peroxidases co-regulated with C3H/APX. One of the significantly downregulated peroxidases, PRX52, is a basic peroxidase that has been linked to lignification. A previous study in Arabidopsis has shown that suppression of AtPRX52 causes a reduction in S-lignin content (Fernandez-Perez et al., 2015). Our results suggest that the markedly reduced levels of several lignin polymerizing enzymes, and particularly PRX52, could be associated with the reduction of S-lignin observed in the C3H/APX-KD lines. CCR proteins are involved in the initial step of monolignol biosynthesis (Lauvergeat et al., 2001) and catalyze the NADPH-dependent conversions of *p*-coumaroyl-CoA, feruloyl-CoA and sinapoyl-CoA into the corresponding aldehydes (Davin et al., 2008; Vanholme et al., 2010). Previous work on tobacco has shown that CCR downregulation reduce plants’ lignin content and increases the S/G ratio (Piquemal et al., 1998; Jones et al., 2001). Our proteomic analysis identified decreased abundance of two CCR-like proteins with unknown roles, and increased abundance of one CCR homolog of *Panicum virgatum* CCR1 with substrate preference for feruloyl CoA over sinapoyl CoA, Caffeoyl CoA and Coumaroyl CoA (Escamilla-Trevino et al., 2010) in the C3H/APX-KD lines, which suggests an efficient conversion of feruloyl-CoA to coniferaldehyde for lignin production in these plants. Coniferaldehyde can be converted to coniferyl alcohol (G-lignin precursor) directly *via* CAD. Therefore, increased abundance of CCR1 could also be partially responsible for reduced S/G ratios seen in the C3H/APX-KD lines. Lastly, proteomics measurement identified PMT, a grass-specific acyltransferase enzyme, to be increased in abundance in C3H/APX-KD plants compared to controls. This protein acylates monolignols with *p*-coumarate prior to their incorporation into lignin (Withers et al., 2012; Petrik et al., 2014). The monolignol conjugates produced by PMT do not enter the radical coupling (polymerization) reactions of lignification (Withers et al., 2012). Brachypodium plants overexpressing PMT have shown to contain less lignin likely due to excessive BdPMT activity drawing *p*-coumaroyl-CoA away from monolignol biosynthesis, thereby lowering the amounts of monolignols available for polymerization (Petrik et al., 2014). Therefore, upregulation of PMT seen in C3H/APX-KD plants compared to controls could have contributed to the lower level of lignin observed in the C3H/APX-KD lines. Changes in other phenylpropanoids, particularly flavonoids, can impact the cellular oxidative stress, auxin movement and plant development (Jacobs and Rubery, 1988; Taylor and Grotewold, 2005; Agati et al., 2012; Brunetti et al., 2013). C3H/APX-KD has been shown to alter the protein abundance of flavonoid biosynthesis pathway which could have a broader impact on plant development and fitness.

Mediator is a large, multi-subunit conserved transcriptional coregulatory complex that is increasingly recognized as an essential element of both basal and regulated eukaryotic transcription (Kim et al., 1994; Kornberg, 2007). In plants, mediators are involved in regulating complex biological processes, including pathogen resistance (Kidd et al., 2009; Wathugala et al., 2012), reproductive development (Imura et al., 2012), and phytochrome signaling (Cerdan and Chory, 2003). Recent studies suggest that Mediator also plays a key role in regulation of phenylpropanoid homeostasis in Arabidopsis (Bonawitz et al., 2012; Bonawitz et al., 2014). In Arabidopsis, the *p-*coumaroyl shikimate 3′-hydroxylase (C3’H) mutation associated lignin deficiency and negative growth phenotype can be overcome by the disruption of the Mediator complex subunits MED5a and MED5b (Bonawitz et al., 2014; Muro-Villanueva et al., 2019). Although MED5a and MED5b were not identified in our dataset, our proteomics analysis identified nine other significantly upregulated subunits of the Mediator complex in C3H/APX-KD compared to controls. In a similar vein, transcription factor families like MYB and WRKY also play crucial roles in regulating various cellular processes including phenylpropanoid synthesis (Yang et al., 2017; Muro-Villanueva et al., 2019; Zhang et al., 2020). A recent study identified an importin-beta-like protein that mediates the transport of the MYB4 transcriptional repressor from the cytosol into the nucleus (Zhao et al., 2007; Panda et al., 2020). In Arabidopsis, importin-beta-like protein and MYB4 have been shown to mediate lignin modification-induced dwarfism, and suppression of these genes alleviates the dwarf phenotype in C3’H mutant but not in CCR1 mutant (Panda et al., 2020). Like Mediator subunits, our proteomics analysis identified several MYB and WRKY transcription factors including MYB4 and importin-beta-like protein, which were all significantly upregulated in C3H/APX-KD compared to controls. Overall, the proteomics analysis of the low lignin Brachypodium C3H/APX-KD indicates that there are complex and subtle transcriptional control mechanisms linking phenylpropanoid biosynthesis, growth and development, and pathogen resistance. Moreover, some of the transcriptional regulators identified in this study can be targets for future studies to rescue the dwarf and non-viable seed phenotype observe in C3H/APX-KD plants.

Phytohormones are biochemical molecules that are known to impact various biological processes in plants including the progression and regulation of the senescence process, and plant growth and development (Fujioka and Yokota, 2003; Thomas and Sun, 2004; Joshi et al., 2019; Guo et al., 2021). Phytohormones can act either close to or remote from their sites of synthesis to regulate responses to environmental stimuli (Fahad et al., 2015). As a response to environmental stress, they can reduce plant growth to focus its resources on withstanding the stress (Fahad et al., 2015). Phytohormones like ethylene, jasmonic acid (JA), salicylic acid (SA) and abscisic acid (ABA) are known to promote leaf senescence; in contrast, cytokinins (CKs), gibberellic acid (GA), and auxin are known to delay leaf senescence (Gan and Amasino, 1995; Gan and Amasino, 1997; Lim et al., 2007; Li et al., 2013; Zhang et al., 2013; Guo et al., 2021). Our proteomics results identified significantly changing proteins involved in biosynthesis and/or perception of all major phytohormones. Therefore, the altered hormonal balance might also contribute towards the negative growth phenotype observed with C3H/APX-KD. Besides phytohormones, the senescence process is also highly coordinated through senescence-associated genes (SAGs). Proteomics and transcriptomics analyses identified SAG proteins/gene which were highly downregulated in C3H/APX-KD compared to controls, and senescence-associated e3 ubiquitin, which prevents premature senescence, as significantly upregulated in C3H/APX-KD compared to controls. The delayed senescence observed in C3H/APX-KD might therefore be influence by the SAGs in addition to phytohormone abundance changes.

C3H/APX is a bifunctional cytosolic ascorbate peroxidase that is known to play crucial roles in regulating the level of cytosolic peroxides *via* ascorbate-glutathione cycle (Davletova et al., 2005). Through this process, cellular ascorbate content and redox state are maintained (Gallie, 2013). Thus, it is plausible that the deficiency of this peroxidase would lead to disruption in cellular redox homeostasis. Proteome results showed the decreased abundance of several proteins involved in the ascorbate-glutathione cycle, supporting the potential disruption in redox balance. In a similar fashion, the proteome results also showed substantial reduction of photosynthesis related proteins such as NAD(P)H-quinone oxidoreductase, serine/threonine-protein kinase STN8, and chlorophyll a-b binding protein in C3H/APX-KD compared to controls. NAD(P)H-quinone oxidoreductase is involved in shuttling electrons from NAD(P)H:plastoquinone to quinones in the photosynthetic electron transport chain (Melo et al., 2004). Serine/threonine-protein kinase STN8 is a light-dependent kinase that specifically phosphorylates N-terminal threonine residues in components of the core antenna complex of photosystem II like psbA/D1, psbD/D2, psbC/CP43 and psbH (Vainonen et al., 2005). Chlorophyll a-b binding protein functions as a light receptor to capture and deliver excitation energy to photosystems (Allen, 1992; Aro and Ohad, 2003). Under normal photosynthetic conditions, cells produce a steady rate of reactive oxygen species which are usually scavenged to prevent cellular oxidative damage (Pnueli et al., 2003). In Arabidopsis, the absence of the cytosolic C3H/APX was shown to collapse the entire chloroplastic H_2_O_2_-scavenging system (Davletova et al., 2005). Thus, it is reasonable that the C3H/APX knockdown of Brachypodium with disrupted redox homeostasis has damaged organelle H_2_O_2_-scavenging systems, which could lead to compromised photosynthetic ability. Moreover, proteomics analysis also showed decreased abundance of stromal and thylakoid membrane bound APX in C3H/APX-KD compared to controls, suggesting reduced ascorbate recycling in the chloroplast that would also compromise plant’s photosynthetic ability. Since the photosynthesis and growth are positively correlated (Kirschbaum, 2011), the potentially compromised photosynthetic ability of Brachypodium C3H/APX-KD might be one of the causes of the negative growth phenotype observed.

Exposure of DNA to reactive species can cause strand breakage, nucleic acid-protein crosslinking, and nucleotide base modifications (Marnett, 2000; Berg et al., 2004). The accumulation of DNA damage could lead to undesirable effects on the growth and yield of plants by blocking critical cellular processes such as transcription or replication and causing cell death (Kimura and Sakaguchi, 2006). Plant cells can activate DNA damage responses to repair damaged DNA; however, this process also contributes to reduction in plant growth (Nisa et al., 2019). Our proteomics results identified a higher abundance of proteins involved in DNA damage response. This implies that C3H/APX-KD has elevated oxidative DNA damage contributing to the negative growth phenotype observed in the C3H/APX-KD knockdown line. Similarly, ROS regulates plant growth through phytohormone signaling (Considine and Foyer, 2014). They can also act as signaling molecules that are perceived by various cellular kinases to trigger the downstream defense signaling pathways (Jalmi and Sinha, 2015). In plants, trade-offs between growth and defense exist and the plant’s optimal fitness depends on growth-defense balance (Ha et al., 2021). Proteome results suggest that the C3H/APX-KD plants, while having reduced growth, has a higher abundance of disease resistance proteins regulated *via* intracellular kinases which could potentially be activated by ROS.

Proteomics result from C3H/APX-KD Brachypodium is further supported by metabolomic measurements of C3H/APX-KD Brachypodium (Barros et al., 2019). In particular, linoleic acid was upregulated by more than 2-fold in C3H/APX-KD compared to WT (**Supplement Table 7**). Linoleic acid has been shown to regulate the expression of genes involved in the abiotic stress response, particularly those mediated by ROS signaling (Mata-Perez et al., 2015). Aconitic acid is another metabolite that was significantly upregulated by more than 2-fold in C3H/APX-KD compared to WT (**Supplement Table 7**). This metabolite has been previously demonstrated to strongly inhibit the soybean (*Glycine max*) plant’s growth and its photosynthetic ability while enhancing the cellular H_2_O_2_ content (Bortolo et al., 2018). Metabolomics also detected a substantial decrease of an ascorbate conjugate metabolite which could be related to ascorbate recycling (**Supplement Table 7**). Moreover, gentisic acid-5-*O*-glucoside was also substantially decreased. This metabolite and its derivatives are known to act as antioxidants and have been associated with plant defense and salicylic acid metabolism (Boreham and Martin, 1969; Todd et al., 1971; Campbell et al., 1984; Joshi et al., 2012).

**Figure 8:**
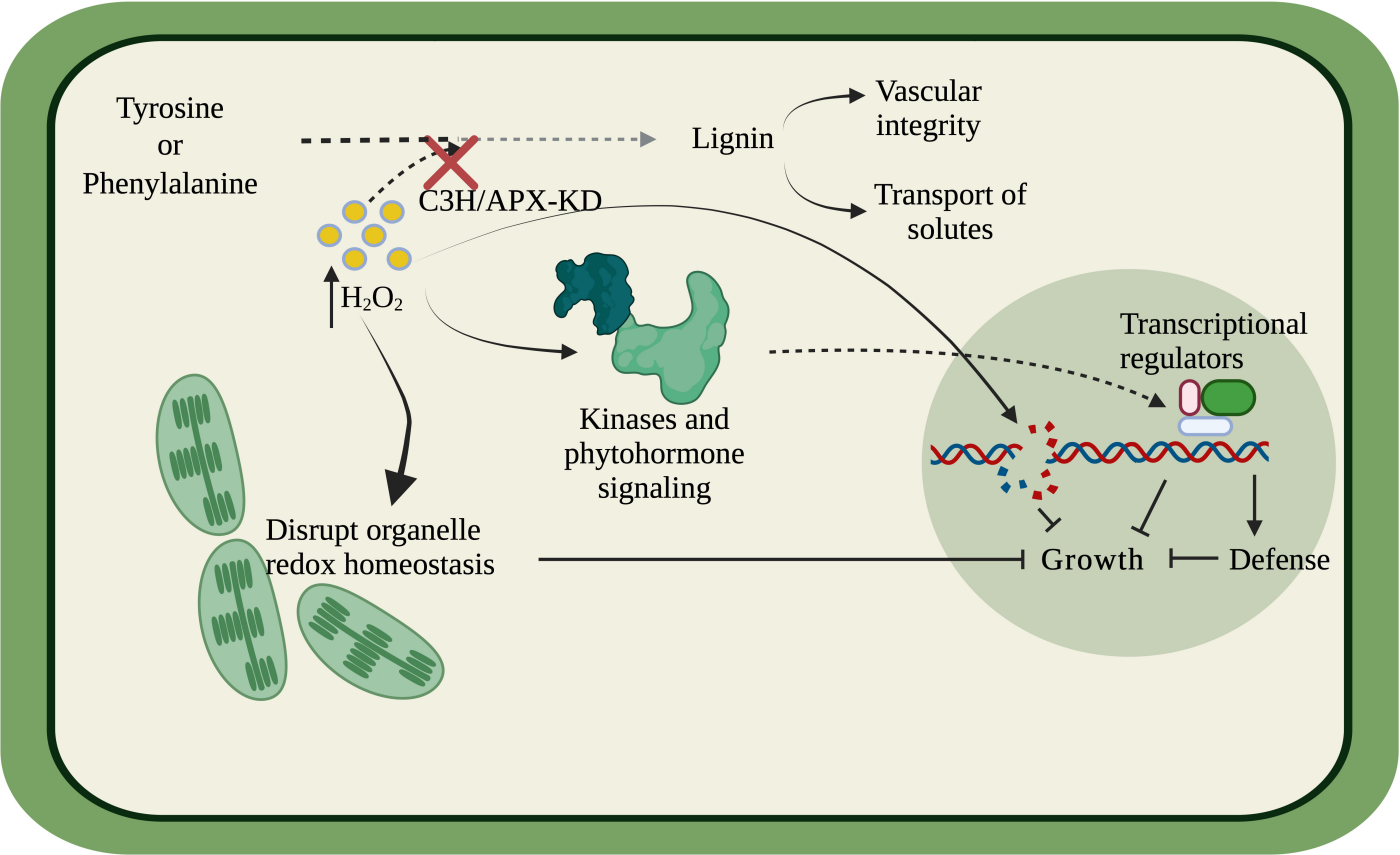
Simplified model linking C3H/APX and the lignin biosynthesis pathway to other biological processes. C3H/APX utilizes hydrogen peroxide (H_2_O_2_) as a co-factor in the lignin biosynthetic pathway. The deficiency of C3H/APX elevated cellular oxidative stress thus disrupting the cellular and organelle redox balance contributing to reduced photosynthetic ability. The redox imbalance can directly elevate DNA damage. Moreover, the disrupted redox homeostasis can transduce stress signal downstream through intracellular kinases or phytohormone-based signaling to regulated growth/defense transcription and translation with the help of transcriptional regulators. The compromised photosynthetic ability, oxidative DNA damage, elevated defense response in tandem could contribute to the negative growth phenotype of the C3H/APX-KD lines.

Overall, our findings suggest that C3H/APX is involved in specific crosstalk between lignin biosynthesis and abiotic stress through ROS metabolism, and thus knockdown of C3H/APX results in a negative growth phenotype through the growth-defense trade-offs (**Figure 8**). The deficiency of C3H/APX likely results in the ROS imbalance, which elevates cellular oxidative stress, thus disrupting the cellular and organelle redox balance. Additionally, the redox imbalance can directly elevate DNA damage, compromise photosynthesis and trigger stress-related signal cascades through intracellular kinases or phytohormone-based signaling to regulate growth/defense transcriptional regulators. Our previous study of lignin-modified plants with growth defects has shown alterations in the carbon-nitrogen balance (Barros et al., 2022). Meanwhile, this study has shown ROS and/or redox imbalance correlate with these growth inhibitory effects. Further studies are needed to determine if aspects of the trade-offs between carbon and nitrogen allocation or ROS imbalance or both are associated with the growth inhibition observed in lignin-modified plants.

## Data availability statement

All proteomics spectral data in this study were deposited at the ProteomeXchange Consortium *via* the MASSIVE repository (https://massive.ucsd.edu/). The ProteomeXchange project identifier is MSV000089074 and the data can be reviewed under the username “CBI_lignin_c3h” and password “C3HLIGNIN”.

## Author contributions

HS conducted the proteomic measurement, analyzed data, and wrote manuscript. YF, JB and RM assisted with ROS and H_2_O_2_ measurements. NE and TT conducted metabolomic measurement. RD and RH contributed to experimental design and assisted with manuscript editing. JB contributed with experimental design, data analysis and manuscript editing. PA assisted proteomic measurements and contributed to data analysis and manuscript editing. All authors contributed to the article and approved the submitted version.

## Funding

This research was sponsored by the Genomic Science Program, U.S. Department of Energy, Office of Science, Biological and Environmental Research, as part of the Center for Bioenergy Innovation (CBI) (https://cbi.ornl.gov/). Oak Ridge National Laboratory is managed by UT-Battelle, LLC, for the U.S. Department of Energy under contract DE-AC05-00OR22725.

## Acknowledgments

We thank Dr Xiaolan Rao for support with the RNA-seq data analyses and Meredith L. Yeary for support with metabolomics data extraction. This manuscript has been authored by UT-Battelle, LLC under Contract No. DE-AC05- 00OR22725 with the U.S. Department of Energy. The United States Government retains and the publisher, by accepting the article for publication, acknowledges that the United States Government retains a non-exclusive, paid-up, irrevocable, worldwide license to publish or reproduce the published form of this manuscript, or allow others to do so, for United States Government purposes. The Department of Energy will provide public access to these results of federally sponsored research in accordance with the DOE Public Access Plan (http://energy.gov/downloads/doe-public-access-plan).

## Conflict of interest

The authors declare that the research was conducted in the absence of any commercial or financial relationships that could be construed as a potential conflict of interest.

## Licenses and permissions

This manuscript has been authored by UT-Battelle, LLC under Contract No. DE-AC05- 00OR22725 with the U.S. Department of Energy. The United States Government retains and the publisher, by accepting the article for publication, acknowledges that the United States Government retains a non-exclusive, paid up, irrevocable, world-wide license to publish, or reproduce the published form of this manuscript, or allow others to do so, for United States Government purposes. The Department of Energy will provide public access to these results of federally sponsored research in accordance with the DOE Public Access Plan (http://energy.gov/downloads/doe-public-access-plan).

## Publisher’s note

All claims expressed in this article are solely those of the authors and do not necessarily represent those of their affiliated organizations, or those of the publisher, the editors and the reviewers. Any product that may be evaluated in this article, or claim that may be made by its manufacturer, is not guaranteed or endorsed by the publisher.
