# Supplementary material for: Multi-omic characterization of bifunctional peroxidase 4-coumarate 3-hydroxylase knockdown in Brachypodium distachyon provides insights into lignin modification-associated pleiotropic effects
Source: Front Plant Sci. 2022 Sep 28;13:908649. doi: 10.3389/fpls.2022.908649 (PMC9554711; doi:10.3389/fpls.2022.908649)
Supplement: Supplementary file 1 [file Presentation_1.pdf]

Supplement Figures

Multi-omic characterization of bifunctional peroxidase 4-coumarate 3-hydroxylase knockdown in *Brachypodium distachyon* provides insights into lignin modification-associated pleiotropic effects

Him K. Shrestha[1,2], Yosef Fichman[3], Nancy L. Engle[1], Timothy J. Tschaplinski[1], Ron Mittler[4], Richard A. Dixon[3], Robert L. Hettich[1], Jaime Barros[3,4*], and Paul E. Abraham[1,*]

[1]Biosciences Division, Oak Ridge National Laboratory, Oak Ridge, TN, United States.
[2]Genome Science and Technology, University of Tennessee-Knoxville, Knoxville, TN, United States.
[3]Division of Plant Sciences and Interdisciplinary Plant Group, University of Missouri, Columbia, MO, United States
[4]BioDiscovery Institute and Department of Biological Sciences, University of North Texas, Denton, TX, United States.

*** Correspondence:**
Dr. Jaime Barros
Email: jaime.barros@missouri.edu

Dr. Paul E. Abraham
Email: abrahampe@ornl.gov

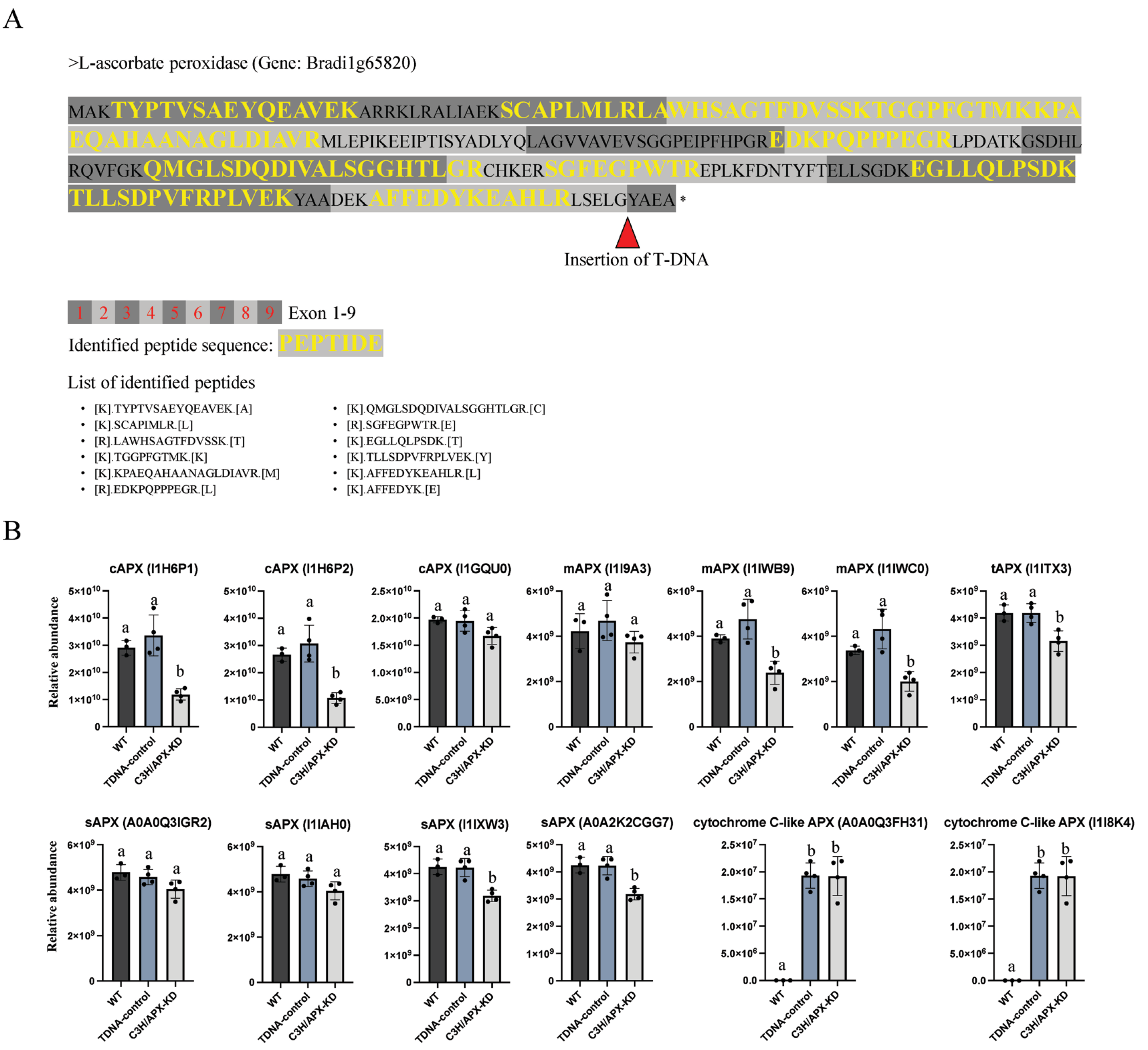

Supp Figure 1: C3H/APX protein coverage and its knockdown impact on cellular ascorbate peroxidases. A) C3H/APX protein coverage from proteomics measurement. Yellow colored letters represent the identified amino acid sequences. The repeating shade of grays shows exon 1 through 9. T-DNA insertion site is shown with red triangle and list of all identified peptides are provided. B) Bar chart shows the effect of targeted knockdown of C3H/APX on other ascorbate peroxidases. Letter above the bar chart represents the binary comparison (ANOVA followed by Tukey HSD) result where the different letters means significant in those conditions. mAPX: microsomal APX, cAPX: cytosolic APX, sAPX: stromal APX, tAPX: thylakoid membrane bound APX.

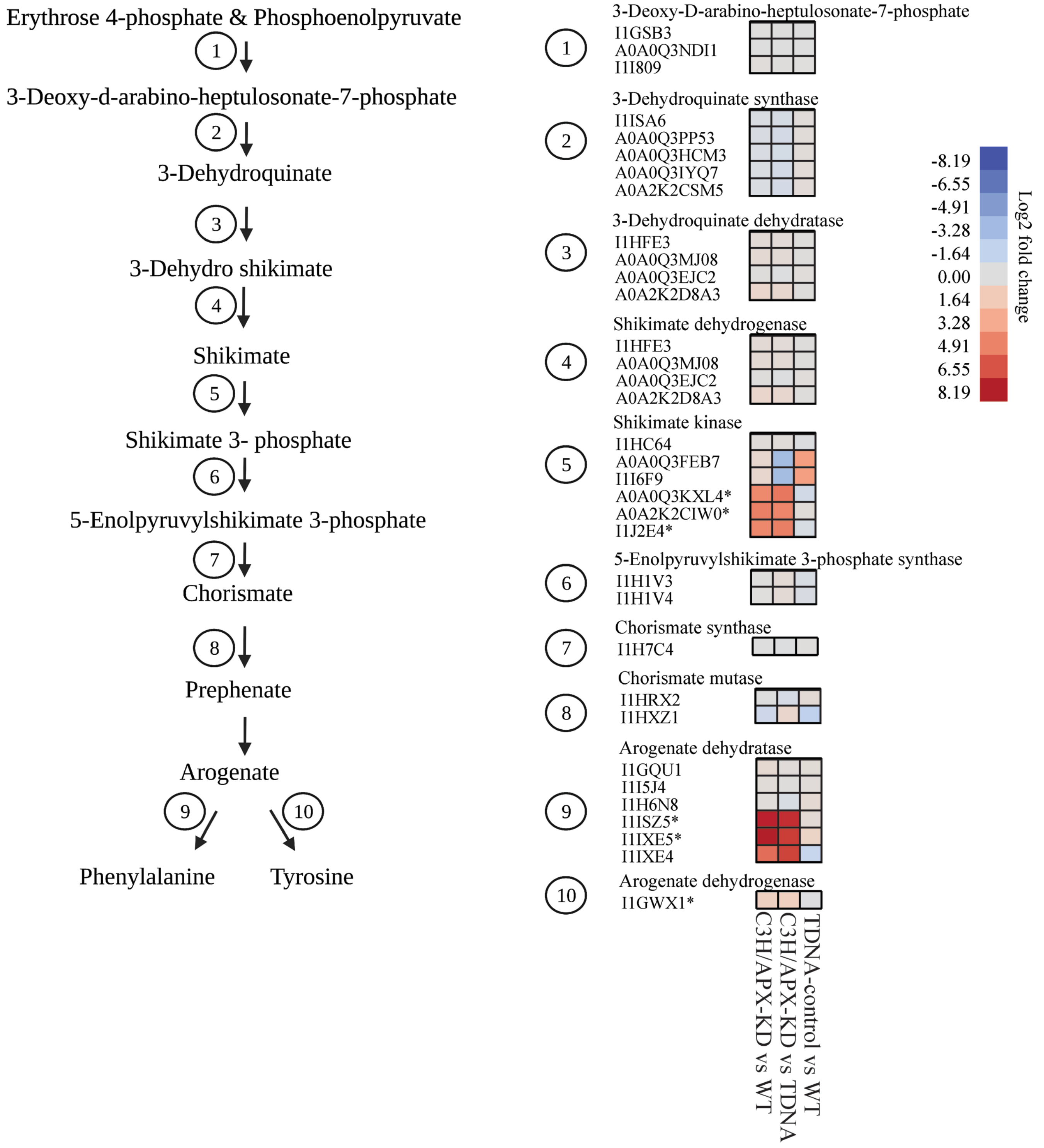

Supp Figure 2: Effect of C3H/APX knockdown on proteins associated with shikimate and aromatic amino acid biosynthesis pathways. Heat cells shows the binary comparison results between experimental conditions and their log2 fold differences. All identified proteins in this pathway are shown. Enzymes significantly changing (adj p-value<0.05) in C3H/APX-KD compared to controls contain * in their accessions.

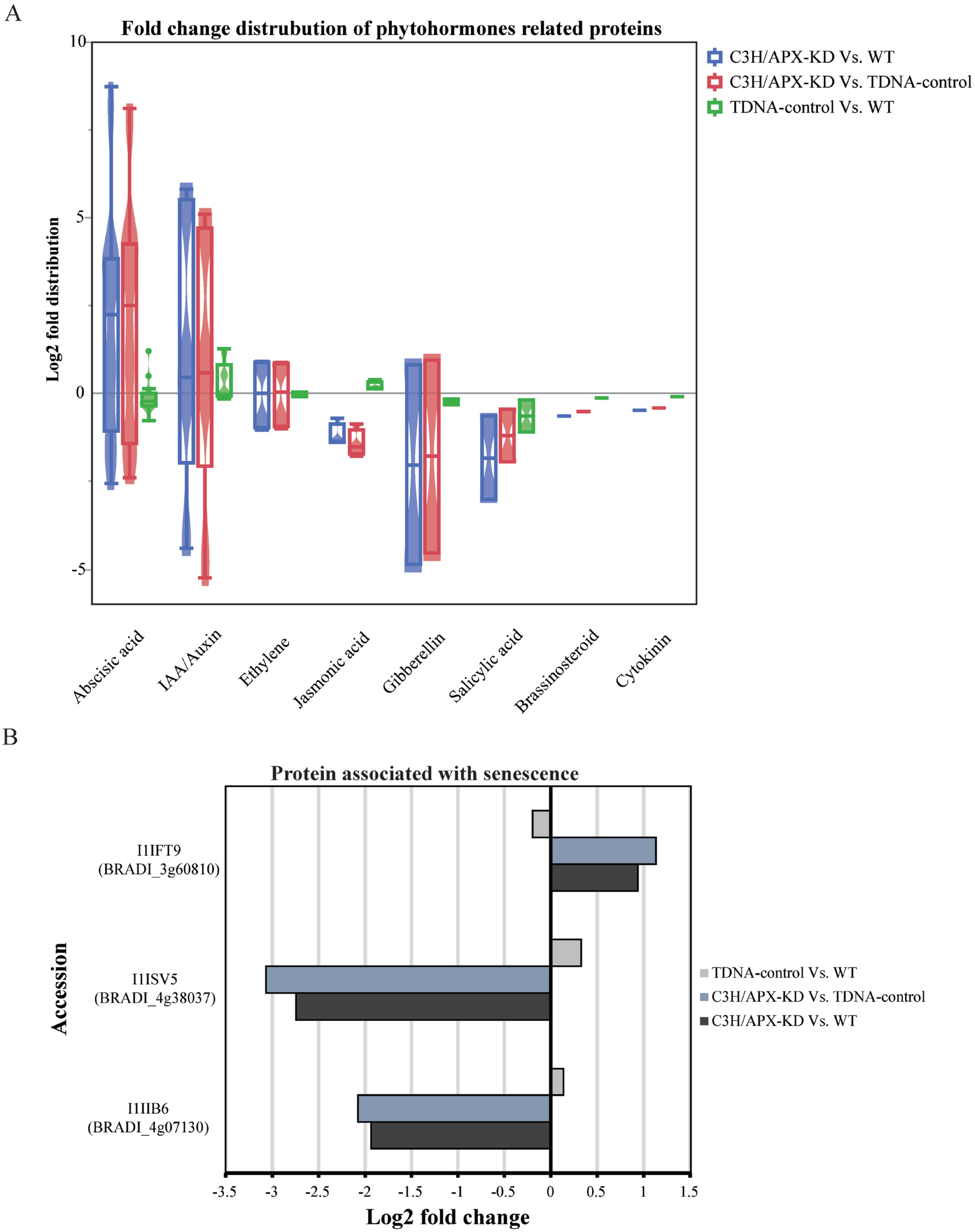

Supp Figure 3: Proteins related to phytohormone perception/biosynthesis and senescence. A) Boxplot and violin plot showing the distribution of log2 fold changes of the proteins involved in phytohormone perception or biosynthesis. All the proteins represented in the figure pass significant threshold of 0.05. Individual protein information is provided in Supplementary table 4. B) Bar chart representing the significantly changing senescence-associated proteins (no. of replicates ≥3). X-axis shows the log2 fold change from the binary comparisons.

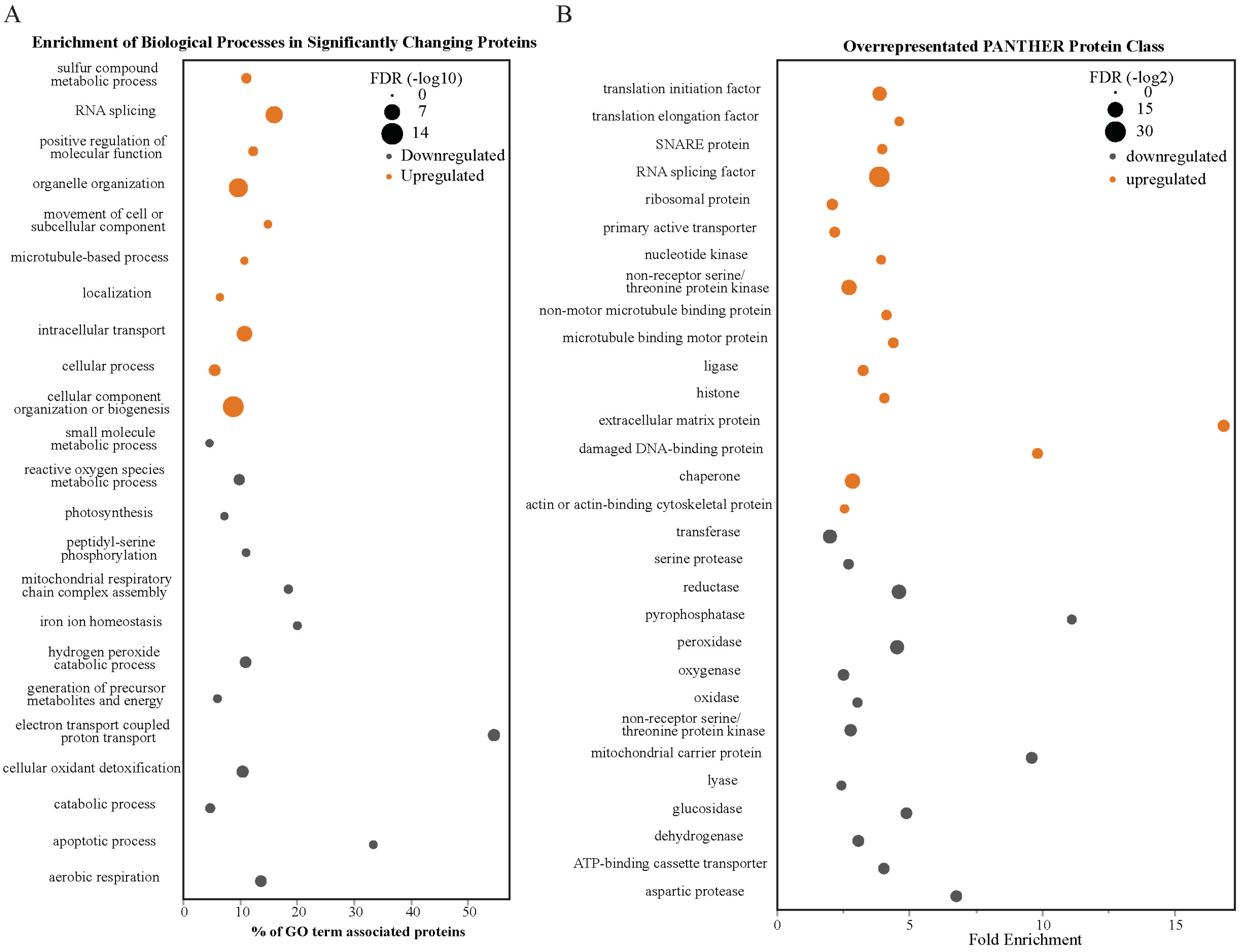

Supp Figure 4: Enriched biological processes and overrepresented protein classes among significantly changing proteins in C3H/APX-KD compared to controls. (A) Bubble plot showing the enriched biological processes among the significantly changing C3H/APX-KD proteins compared to control. GO enrichment was done in g:profiler and GO redundancy was filtered using REVIGO. (B) Bubble plot showing overrepresented protein classes among the significantly changing C3H/APX-KD proteins compared to control. Protein Class overrepresentation test was performed in PANTHER. All the biological processes and protein classes represented passed FDR threshold of 0.05. Bubble size represents the FDR value in -log10 scale (Panel A) and in –log2 scale (Panel B). Bubble color represented up or downregulation in C3H/APX-KD compared to controls.

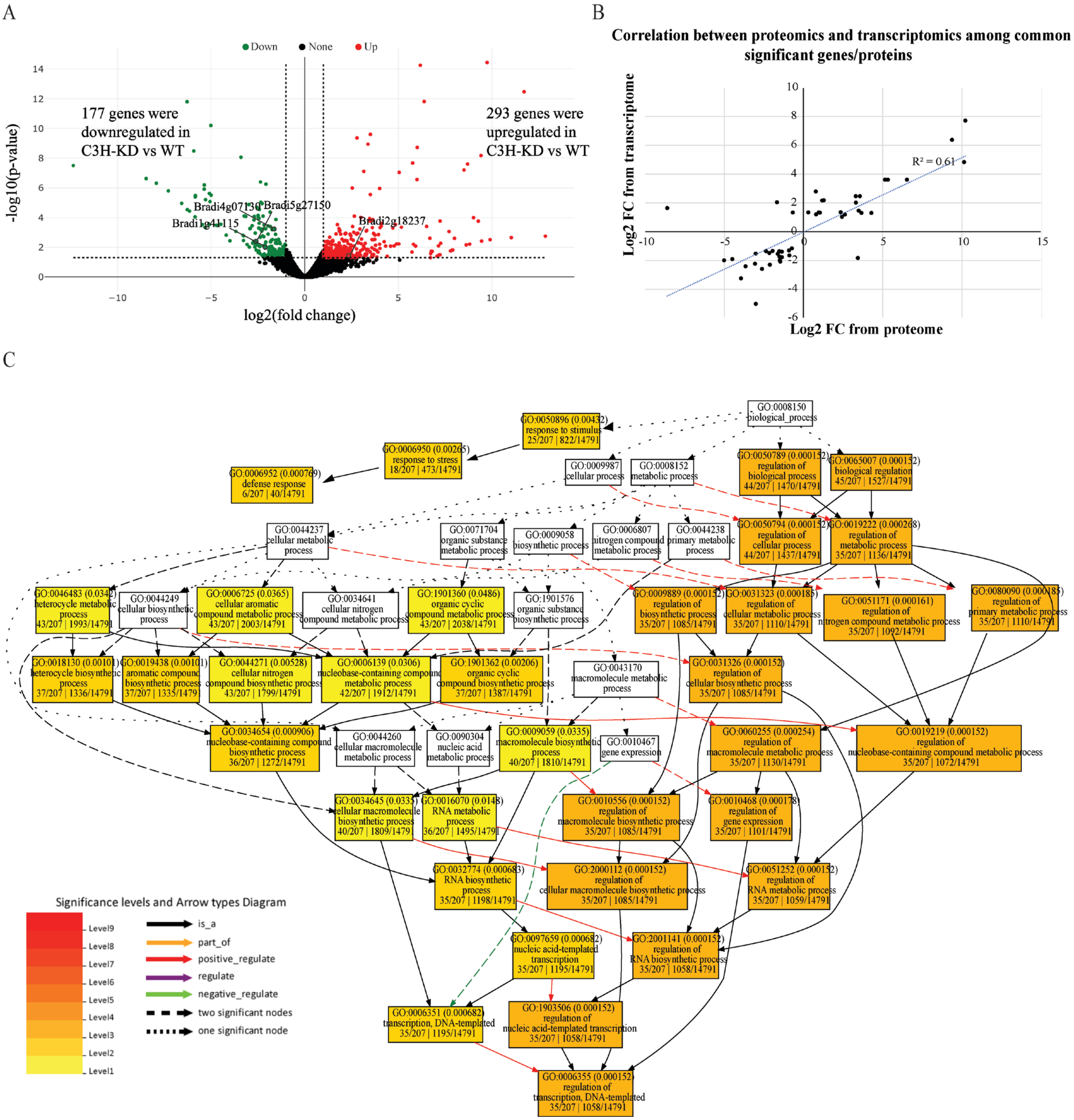

Supp Figure 5: A global view of transcriptional changes between C3H-KD compared to WT Brachypodium. A) Volcano plot showing differentially expressed genes in C3H-KD compared to WT. Red dots are the genes significantly upregulated and green dots are the genes significantly downregulated in C3H-KD compared to WT. Black dots are the genes not significantly changing. Few genes with significant protein changes (pathogen-associated molecular patterns-induced protein: Bradi2g18237; senescence related protein SAG12: Bradi4g07130; peroxidase 12: Bradi5g27150 and peroxidase 52: Bradi1g41115) are labeled in volcano plot. B) Correlation plot of genes and proteins that are significantly changing in both transcriptome and proteome data. R^2 value represent correlation value. C) GO-biological processes enrichment of differentially expressed genes using tool agriGOv2. The color gradient in box shows significance level and arrow shows regulation type.
